# Supplementary material for: Nano colonies: Rearing honey bee queens and their offspring in small laboratory arenas
Source: Heliyon. 2025 Jan 16;11(2):e42042. doi: 10.1016/j.heliyon.2025.e42042 (PMC11791117; doi:10.1016/j.heliyon.2025.e42042)
Supplement: Multimedia component 1 [file mmc1.docx]

| **Component** | **Volume (ml)** |
| --- | --- |
| Amino acid mix* | 20 |
| Lecithin 8% SDS | 12.50 |
| Lemon grass oil | 0.5 |
| Flax seed oil | 3.0 |
| Safflower oil | 1.5 |
| Sea salt | 0.5 (grams) |
| Sugar | 962.0 |
| **Total** | **1000** |
| *see table 2 for components of mixture | |

**Table 1 Components of Supplementary Diet**

**Table 2 Amino Acid mix of Supplementary Diet**

| **Amino Acid** | **(g/1000 ml)** | **Molarity (mM)** |
| --- | --- | --- |
| Arginine | 10.45 | 60 |
| Histidine | 4.65 | 30 |
| Lysine | 8.77 | 60 |
| Tryptophan | 4.08 | 20 |
| Phenylalanine | 8.26 | 50 |
| Methionine | 4.48 | 30 |
| Threonine | 7.15 | 60 |
| Leucine | 11.81 | 90 |
| Isoleucine | 10.49 | 80 |
| Valine | 9.37 | 80 |
| Alanine | 5.34 | 60 |
| Aspartic Acid | 7.99 | 60 |
| Asparagine | 7.93 | 60 |
| Cysteine | 7.27 | 60 |
| Glutamine | 8.77 | 60 |
| Glutamic acid | 8.83 | 60 |
| Glycine | 4.50 | 60 |
| Proline | 6.91 | 60 |
| Serine | 6.31 | 60 |
| Tyrosine | 0.51 | 0.5 |
| **Total** | **143.87** |  |
