# Supplementary material for: Nano colonies: Rearing honey bee queens and their offspring in small laboratory arenas
Source: Heliyon. 2025 Jan 16;11(2):e42042. doi: 10.1016/j.heliyon.2025.e42042 (PMC11791117; doi:10.1016/j.heliyon.2025.e42042)
Supplement: Multimedia component 2 [file mmc2.pdf]

Step by step guide to constructing nano cages (1/5)

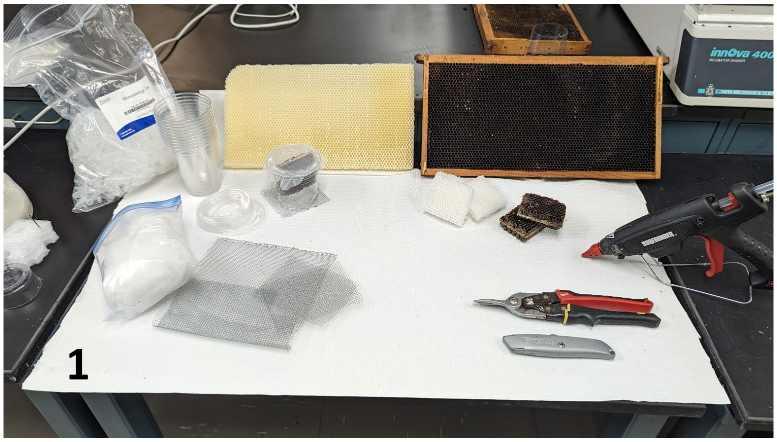

1. The materials needed to construct nano colonies.

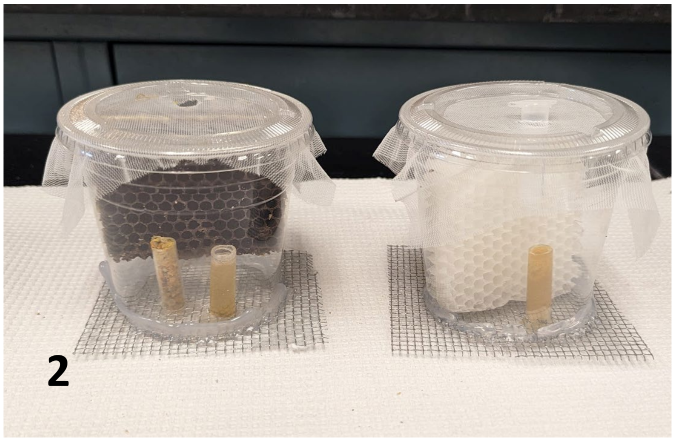

2. Two constructed nano colonies. (**Left)** Made with real comb and two feeders. This is used for queen-right nanos. **(Right)** Made with paraffin wax and a single feeder for a queen-rearing nano.

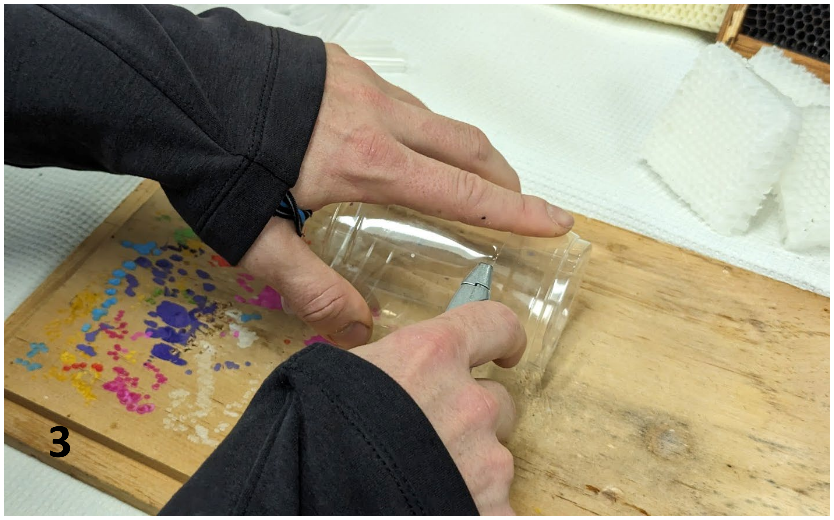

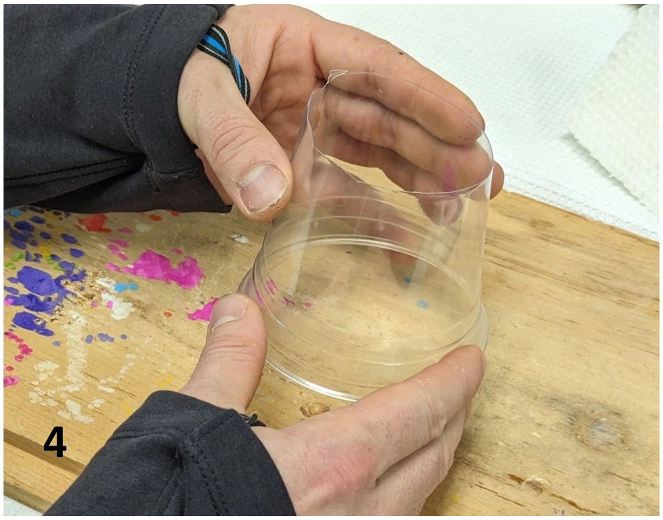

3-4. Preparing the cage. A 16 oz plastic cup is portioned with a razor blade. The cup is pierced with the tip of the blade, and then the cup is rolled until the cup is severed. In picture **(4)** the final cut can be seen.

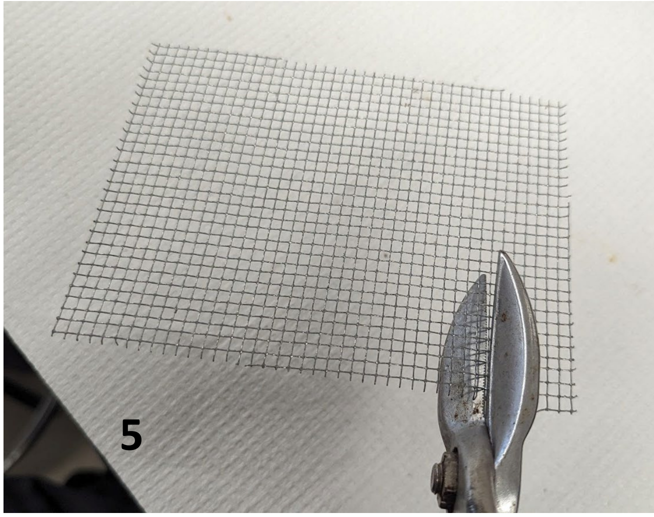

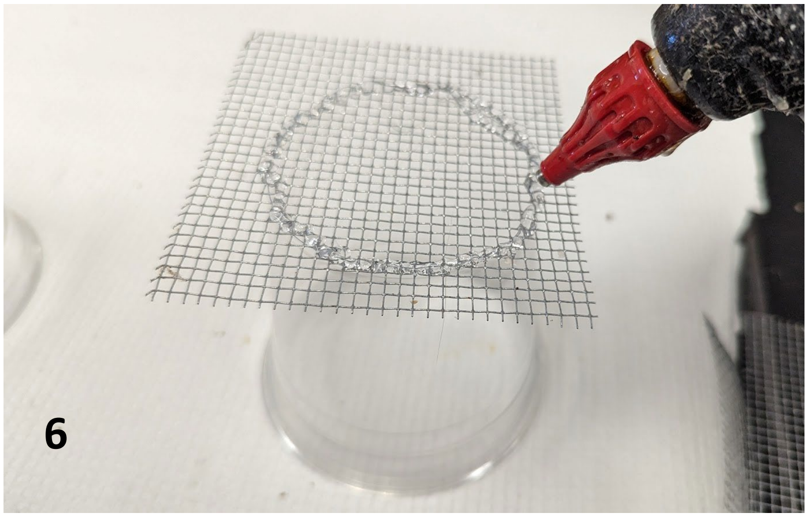

5-6. The bottom of the cage is constructed from #8 hardware cloth. "Tin snips" are an excellent tool to cut pieces to length **(5).** The pieces can then initially be adhered to the cut plastic cups using hot glue **(6).**

Step by step guide to constructing nano cages (2/5)

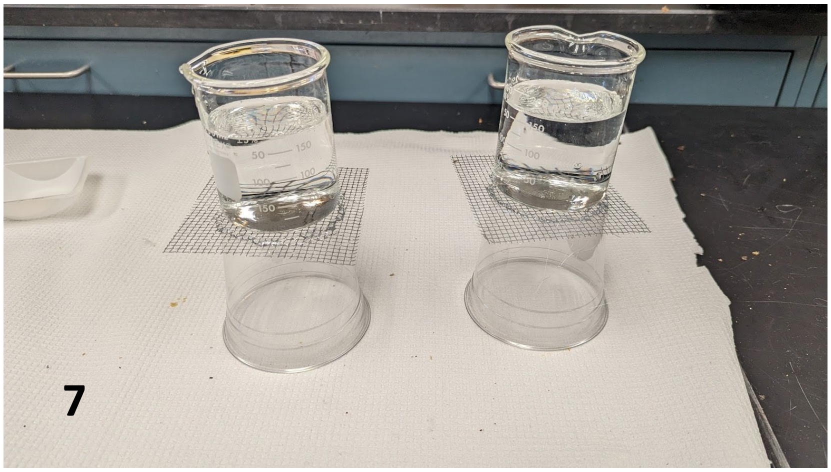

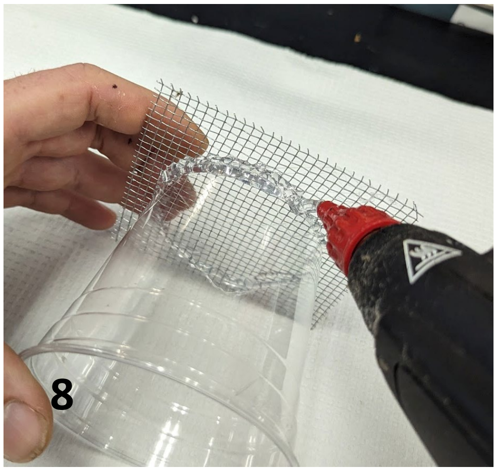

7-8. Weighted beakers ensure good contact between the plastic cup and the hardware mesh while the glue is drying **(7).** Afterwards, the beakers are removed and a bead of hot glue is applied directly on the joint of the hardware cloth and plastic cup. Then leave the cup to dry.

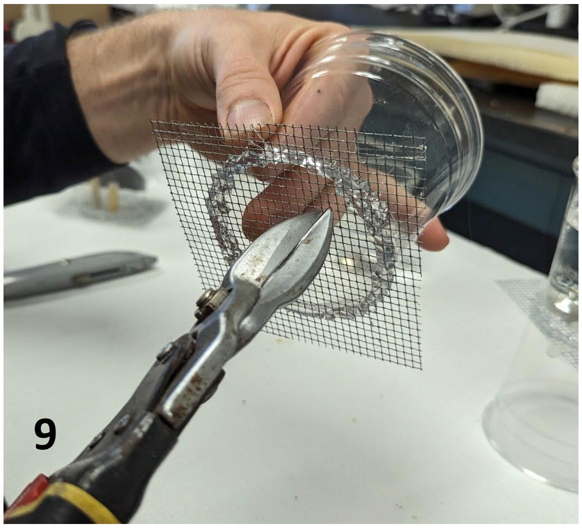

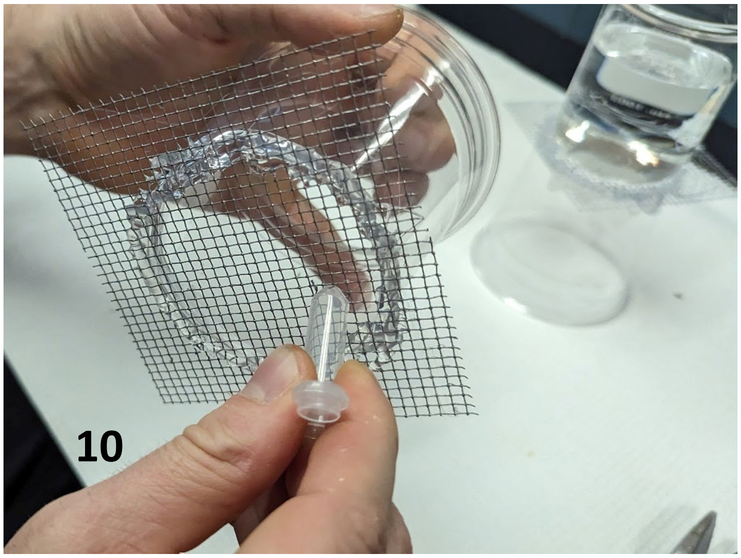

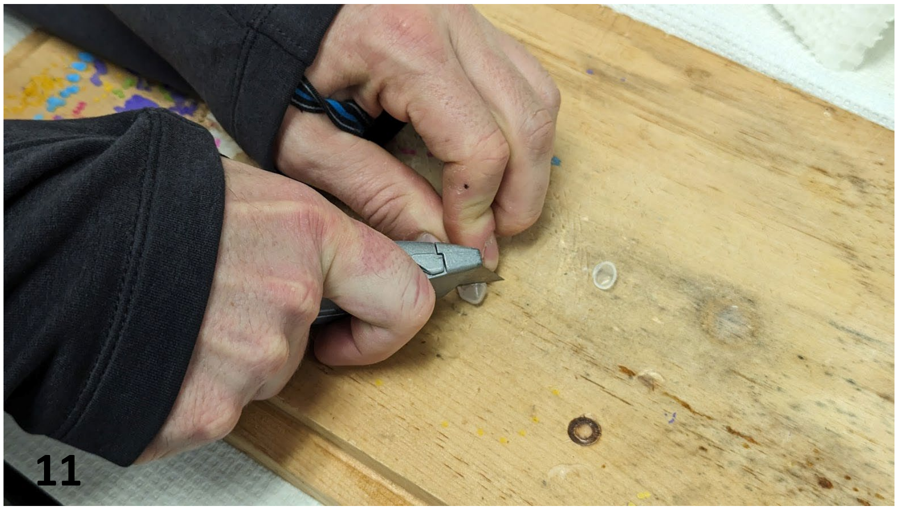

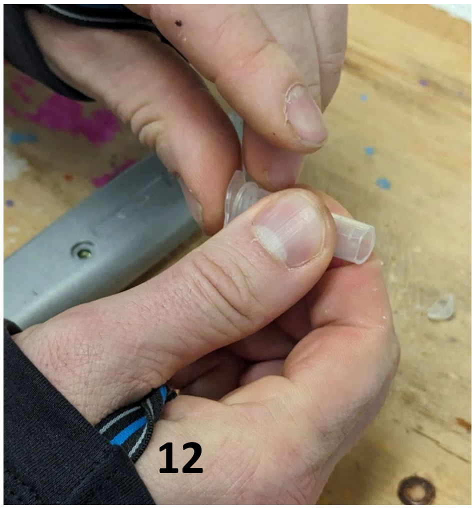

9-12. Access holes for the feeders must be added through the bottom. This can be accomplished with a Dremel tool or **(9)** by using a pair “Tin snips”. The access hole is cut slightly smaller than the diameter of a 2ml Eppendorf tube. A tube is then inserted into the precut hole **(10)** to finish. Making the feeders can be done by cutting off the bottom of a 2 ml Eppendorf tube with a razor blade against a cutting surface **(11-12).**

Step by step guide to constructing nano cages (3/5)

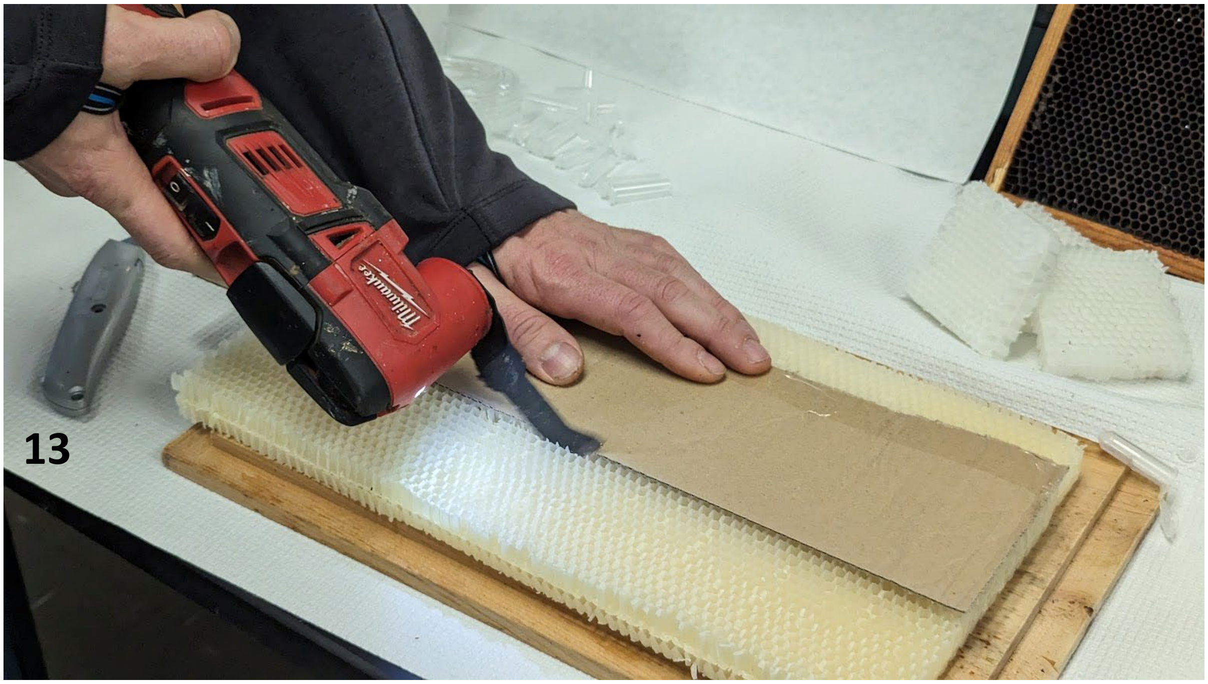

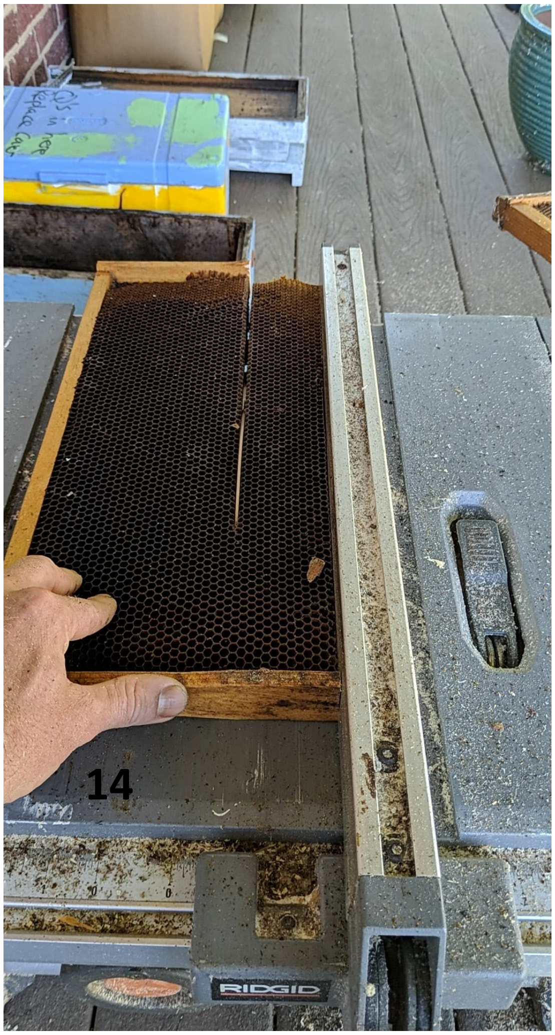

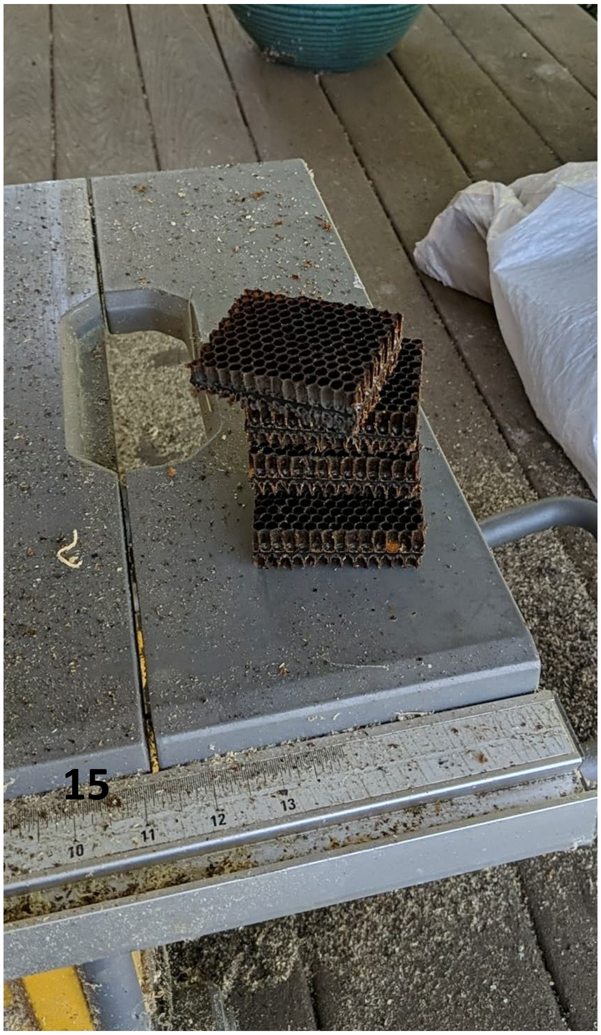

13. Sheets of paraffin wax can be easily cut to length using a “multi-tool” and pre-cut cardboard templates. **14-15.** Frames of wax are cut by first chilling them in a -20c freezer, and then cutting them to length and width on a table saw. **15** Pieces ready to be inserted into prepared cages.

Step by step guide to constructing nano cages (4/5)

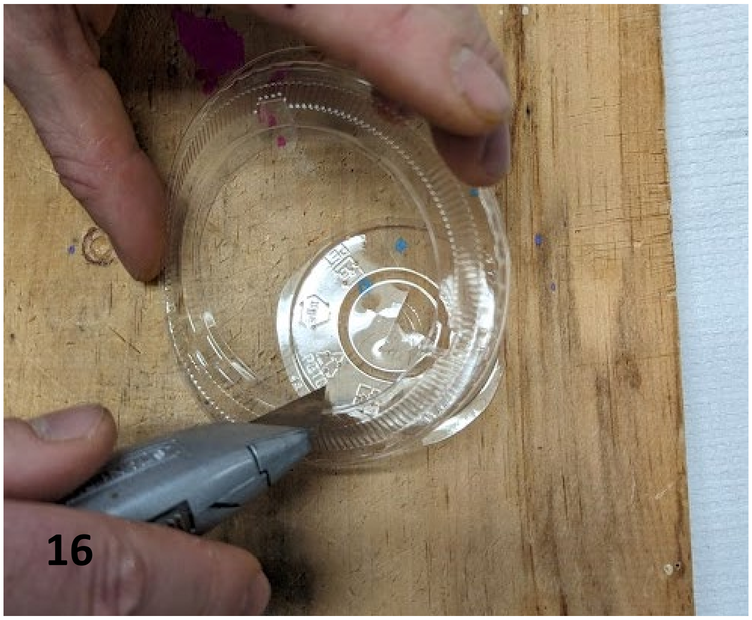

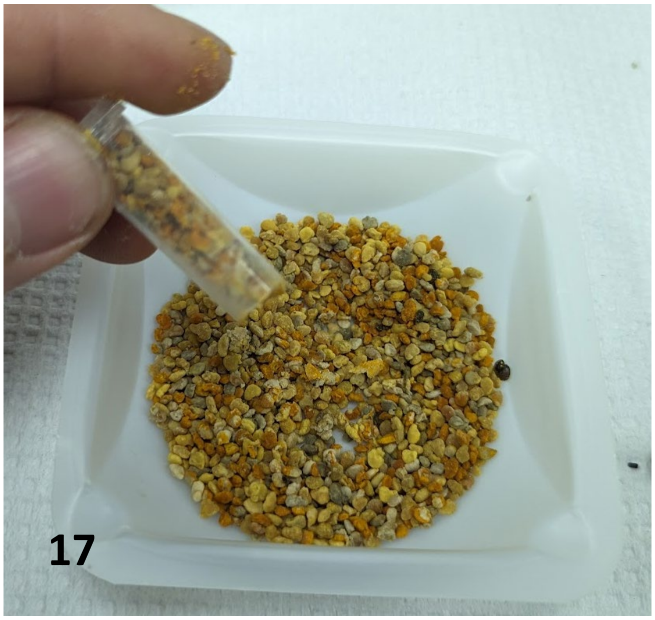

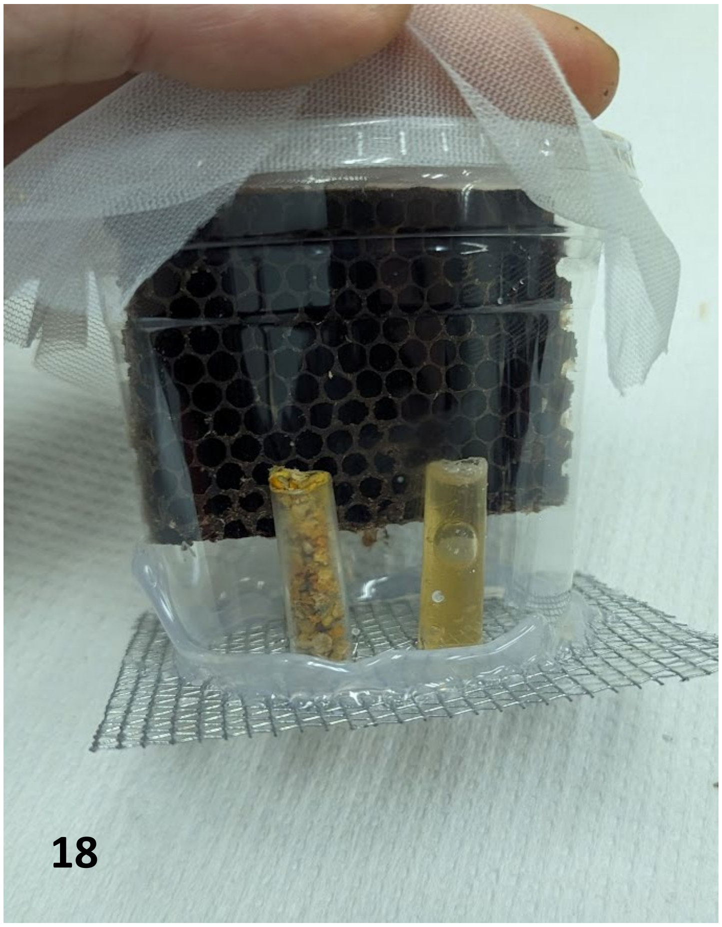

16-18. The lids **(16)** function to seal the noseeum netting. Lids are prepared by piercing a plastic cup lid with a razor blade so that the tip of the razor is lightly imbedded into the cutting board. Then the plastic lid is turned until the blade has completely severed the inside of the lid, leaving just a ring. To finish construction of the cups, a lid is secured in place **(17).** Pollen is loosely packed into a prepared feeder **(18).**

Step by step guide to constructing nano cages (5/5)

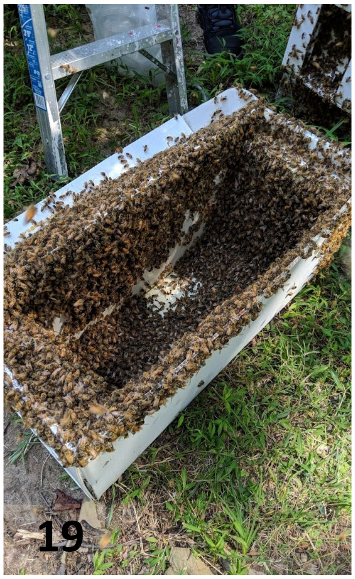

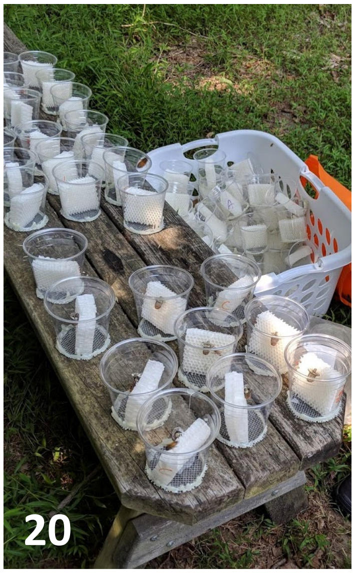

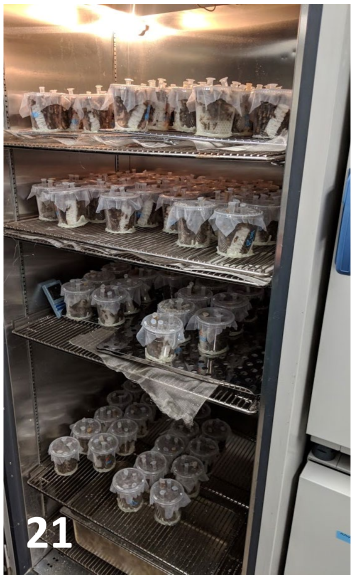

19 - 21. Bulk bees from a cell builder **(19)** are scooped into premade nano setups which already have a started queen cell **(20).** The queen-rearing nanos about one hour after setup in an incubator **(21).**

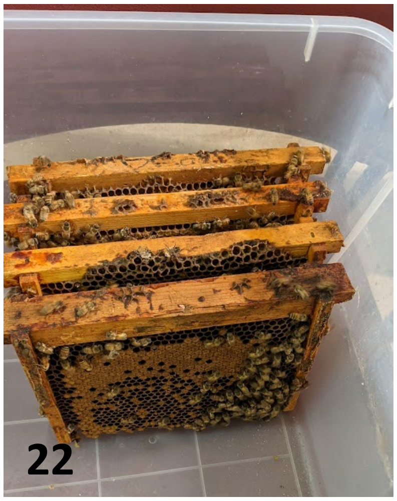

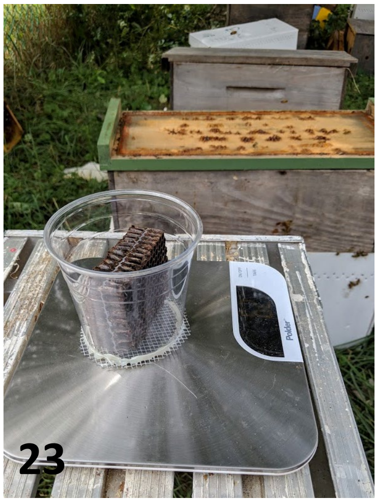

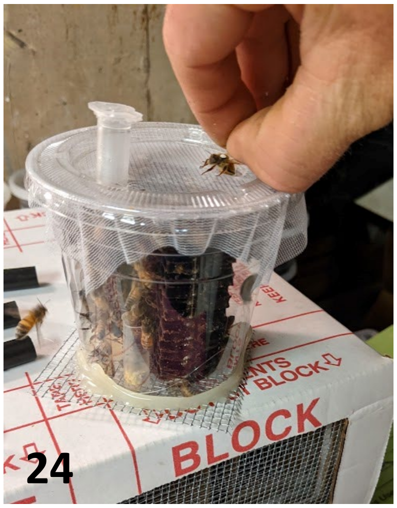

22-25. Newly emerged bees are collected in bulk, and then scooped into premade nano colonies. A scale can be used for quality control **(23)**. Queens can be directly introduced to newly emerge bee nanos through the top entrance **(24).** Replacing feeders and removing dead bees **(25).**

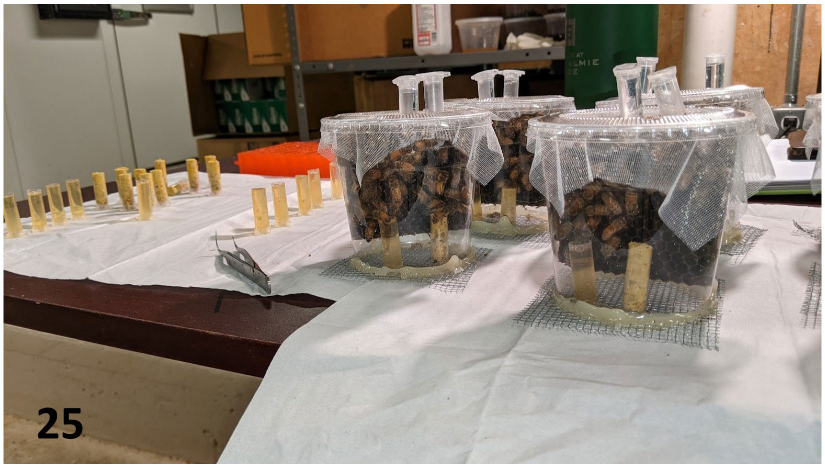

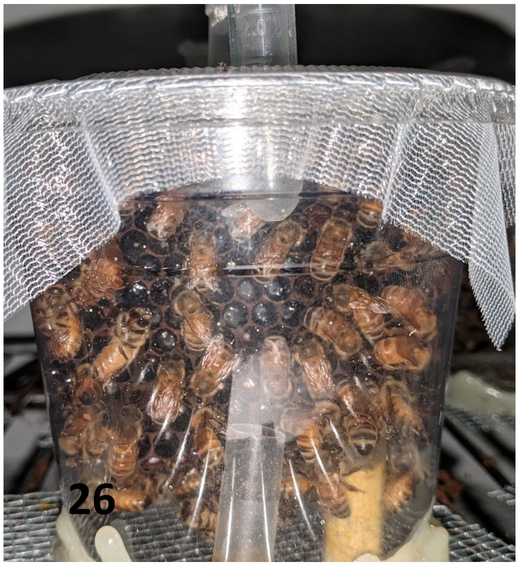

26. A nano colony 8 days after being set up. A bee is consuming pollen through the feeder while others linger or attend brood. Developing larvae are visible through the concave structure of the cage.
